# Pharmacologic or genetic interference with atrogene signaling protects against glucocorticoid-induced musculoskeletal and cardiac disease

**DOI:** 10.1172/jci.insight.182664

**Published:** 2024-11-08

**Authors:** Amy Y. Sato, Meloney Cregor, Kevin McAndrews, Charles A. Schurman, Eric Schaible, Jennifer Shutter, Punit Vyas, Bhawana Adhikari, Monte S. Willis, Marjan Boerma, Tamara Alliston, Teresita Bellido

**Affiliations:** 1 Department of Physiology and Cell Biology, University of Arkansas for Medical Sciences, Little Rock, Arkansas, USA.; 2 Department of Anatomy, Cell Biology, and Physiology, Indiana University School of Medicine, Indianapolis, Indiana, USA.; 3 Department of Orthopaedic Surgery, University of California San Francisco, San Francisco, California, USA.; 4 Advanced Light Source, Lawrence Berkeley National Laboratory, Berkeley, California, USA.; 5 Allegheny Health Network, Pathology and Laboratory Medicine Institute, Pittsburgh, Pennsylvania, USA.; 6 Quest Diagnostics Inc., NE Regional Core Lab, Clifton, New Jersey, USA.; 7 Department of Pathology and Laboratory Medicine, Indiana University School of Medicine, Indianapolis, Indiana, USA.; 8 Department of Pharmaceutical Sciences, University of Arkansas for Medical Sciences, Little Rock, Arkansas, USA.; 9 Department of Medicine, Division of Endocrinology, Indiana University School of Medicine, Indianapolis, Indiana, USA.; 10 Central Arkansas Veterans Healthcare System, Little Rock, Arkansas, USA.; 11 Richard L. Roudebush Veterans Affairs Medical Center, Indianapolis, Indiana, USA.

**Keywords:** Bone biology, Bone disease, Mouse models, Muscle

## Abstract

Despite their beneficial actions as immunosuppressants, glucocorticoids (GC) have devastating effects on the musculoskeletal and cardiac systems, as long-term treated patients exhibit high incidence of falls, bone fractures, and cardiovascular events. Herein, we show that GC upregulate simultaneously in bone, skeletal muscle, and the heart the expression of E3 ubiquitin ligases (atrogenes), known to stimulate the proteasomal degradation of proteins. Activation of vitamin D receptor (VDR) signaling with the VDR ligands calcitriol or eldecalcitol prevented GC-induced atrogene upregulation in vivo and ex vivo in bone/muscle organ cultures and preserved tissue structure/mass and function of the 3 tissues in vivo. Direct pharmacologic inhibition of the proteasome with carfilzomib also conferred musculoskeletal protection. Genetic loss of the atrogene MuRF1-mediated protein ubiquitination in ΔRING mice afforded temporary or sustained protection from GC excess in bone or skeletal and heart muscle. We concluded that the atrogene pathway downstream of MuRF1 underlies GC action in bone, muscle, and the heart, and it can be pharmacologically or genetically targeted to confer protection against the damaging actions of GC simultaneously in the 3 tissues.

## Abstract 2

This study shows that interfering with a common atrophy pathway prevents the harmful actions of glucocorticoids in three tissues: bone, muscle, and the heart.

## Introduction

Glucocorticoids (GC) are commonly used as immunosuppressants to manage a wide range of afflictions, including rheumatoid arthritis, asthma/pulmonary diseases, autoimmune diseases, and organ transplantation and are frequently included in cancer chemotherapy regimens, resulting in millions of patients treated with GC worldwide (1–3). In the last decades, GC usage has grown by an estimated 14%–34% (4, 5), and currently, 4 million patients in the US (6) and 15 million patients in Europe (3–5, 7) are receiving GC. Long-term GC treatment leads to a chronic state of excess, which adversely affects multiple tissues, including the musculoskeletal and cardiac systems, with the consequent increase in bone fractures and cardiovascular events (2, 8–11). Furthermore, the healthcare costs associated with these GC side effects total billions of dollars per year in the US, independently of the primary disease (2, 12). Thus, identification of potential targetable pathways mediating the iatrogenic side effects of GC is needed.

A common feature of chronic GC excess in bone, skeletal muscle, and the heart is the structural deterioration and/or loss of mass followed by tissue dysfunction. Trabecular and cortical bone thinning and loss of mineral density are hallmarks of GC-induced bone disease (13–16). Decreased myofiber diameter and loss of mass characterize GC-induced sarcopenia (14), whereas thinning of the left ventricular wall followed by eccentric hypertrophy marks the early phase of cardiovascular disease (CVD) induced by GC (17). The obvious differences among the tissues notwithstanding, this evidence suggests that a common pathway regulating tissue mass and structure underlies GC action in these organs, providing an opportunity for a single protective intervention.

Atrogenes are limiting factors in the rate of proteosomal activity, as increased atrogene expression promotes protein degradation whereas decreased expression lowers proteasomal proteolysis (18–21). Earlier studies showed that GC increase the expression of E3 ubiquitin ligases (atrogenes) that label proteins for proteasomal degradation in skeletal muscle (18, 19) and bone (14). Moreover, the genetic loss of the atrogene MuRF1 protects against GC actions in skeletal and cardiac tissue (17, 18) and from the bone loss induced by unloading (22).

Prompted by these pieces of evidence, we investigated whether interfering with the atrogene pathway protected simultaneously bone, skeletal muscle, and the heart from structural deterioration and tissue dysfunction using a preclinical mouse model of GC excess. We found that treatment with ligands of the receptor for vitamin D_3_, a hormone with recognized clinical benefits on both musculoskeletal and cardiac systems (23, 24), prevented atrogene increases and GC-induced disease in all tissues. In addition, direct proteasome targeting with the FDA-approved proteosomal inhibitor carfilzomib (carfil; Kyprolis) blocked bone and muscle atrophy induced by GC. Moreover, genetic loss of atrogene MuRF1-mediated protein ubiquitination preserved skeletal and cardiac muscle function and temporarily protected the skeleton against GC effects.

These findings support the notion that pathologic GC actions are mediated by atrogene MuRF1-stimulated proteolysis and that targeting this common pathway either by pharmacologic or genetic means prevents structural deterioration and dysfunction by GC in the musculoskeletal and cardiac systems.

## Results

### VDR ligands block GC upregulation of atrogene expression in bone, skeletal muscle, and cardiac muscle.

**Figure 1:**
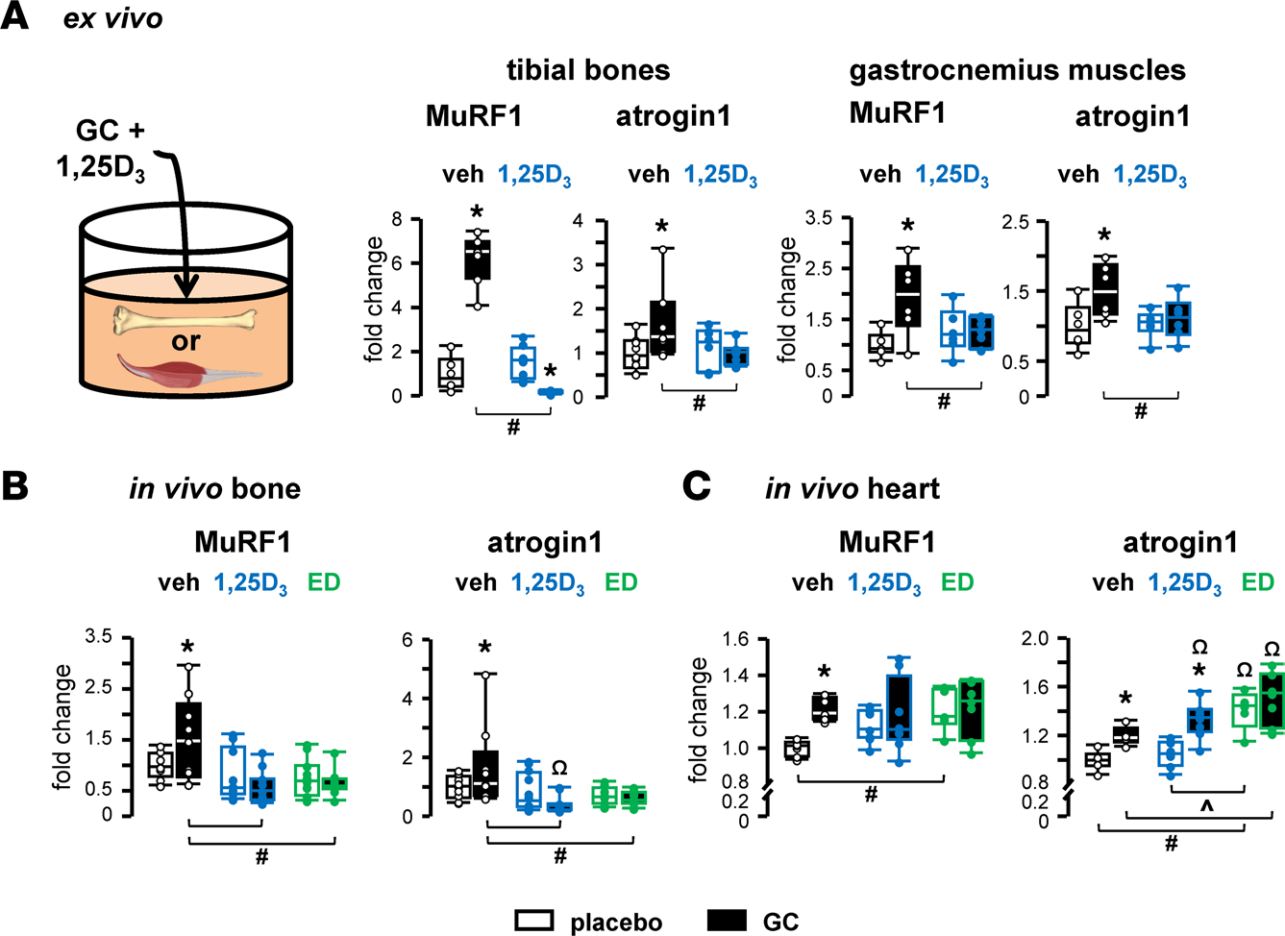
VDR ligands prevent increased atrogene expression by glucocorticoids in bone, muscle, and the heart. *MuRF1* and *Atrogin1* expression was quantified by qPCR in tissues treated without or with GC and vehicle (black outline), 1,25D_3_ (blue outline), or ED (green outline) for 6 hours ex vivo (**A**) or 8 weeks in vivo (**B** and **C**). Mice were implanted with 2.1 mg/kg/d prednisolone or placebo slow-release pellets and gavaged 5 times per week with 50 ng/kg/d 1,25D_3_, ED, or vehicle. For ex vivo, *n* = 6–9 bones and *n* = 6–7 for muscles. For in vivo, *n* = 8–11 bones and *n* = 5–8 hearts. *P < 0.05 vs. corresponding controls, ^#^*P* < 0.05 vs. corresponding vehicle treated, ^*P* < 0.05 vs. corresponding 1,25D_3_ treated by 2-way ANOVA, Tukey’s post hoc test, ^Ω^*P* < 0.05 vs. placebo and vehicle-treated controls by 1-way ANOVA, Dunnett’s Method post hoc test.

In bone, GC increase the expression of atrogenes *MuRF1* and *Atrogin1* in ex vivo bone organ cultures and in vivo (Figure 1, A and B), as previously published (14). The expression level of these atrogenes was not altered by 1,25-dihydroxyvitamin D_3_ (1,25D_3_; also known as calcitriol) ex vivo or in vivo or by 2β-(3-hydroxypropyloxy)-1,25-dihydroxyvitamin D_3_ (ED; also known as eldecalcitol) in vivo. However, 1,25D_3_ prevented GC stimulation of atrogene expression ex vivo, and both metabolites blocked GC upregulation of these atrogenes in vivo.

GC also increased atrogene expression in ex vivo cultures of skeletal muscles, as previously published (14), and similarly to the skeletal protection, 1,25D_3_ blocked the GC increase in *MuRF1* and *Atrogin1* expression. Taken together, these findings suggest that the musculoskeletal protection by VDR ligands may occur via suppression of GC-induced atrogene signaling.

In the heart, *MuRF1* and *Atrogin1* expression was also increased by GC, and 1,25D_3_/ED blocked *MuRF1* increases by GC, but not *Atrogin1* (Figure 1C). Overall, 1,25D_3_ and ED by themselves did not alter *MuRF1* expression and *Atrogin1* expression was also increased by ED alone, although no functional consequences were detected in vivo (see below).

Whether 1,25D_3_ and ED altered expression of other known GC target genes was also investigated. The following genes were selected for bone: bone sialoprotein (*Spp1*), runt-related transcription factor 2 (*Runx2*), and collagen 1a1 (*Col1a1*). The following genes were selected for muscle: forkhead box O1 (*Foxo1*), forkhead box O3 (*Foxo3*), and inositol 1,4,5-triphosphate receptor 1 (*Itpr1*). The following genes were selected for the heart: angiotensin II receptor, type 1a (*Agtr1a*); angiotensin II receptor, type 1b (*Agtr1b*); and Kruppel-like transcription factor 15 (*Klf15*). None of these GC target genes were changed by 1,25D_3_ and ED (data not shown). Additionally, the VDR ligands did not uniformly alter *Nr3c1* (GR) expression in these different tissues (data not shown). In muscle and bone, 1,25D_3_ decreased GR expression only in GC-treated mice whereas ED induced no changes either alone or in combination with GC. In the heart, the VDR ligands did not affect GR expression either alone or in combination with GC. Therefore, in summary, these findings suggest that prevention of the GC-induced increase in atrogene expression by the VDR ligands seems to be not due to a generalized effect on all GC target genes and that it is independent of changes in GR expression.

### Ligands of the VDR protect from bone loss and microarchitecture deterioration by blocking GC effects on remodeling.

**Figure 2:**
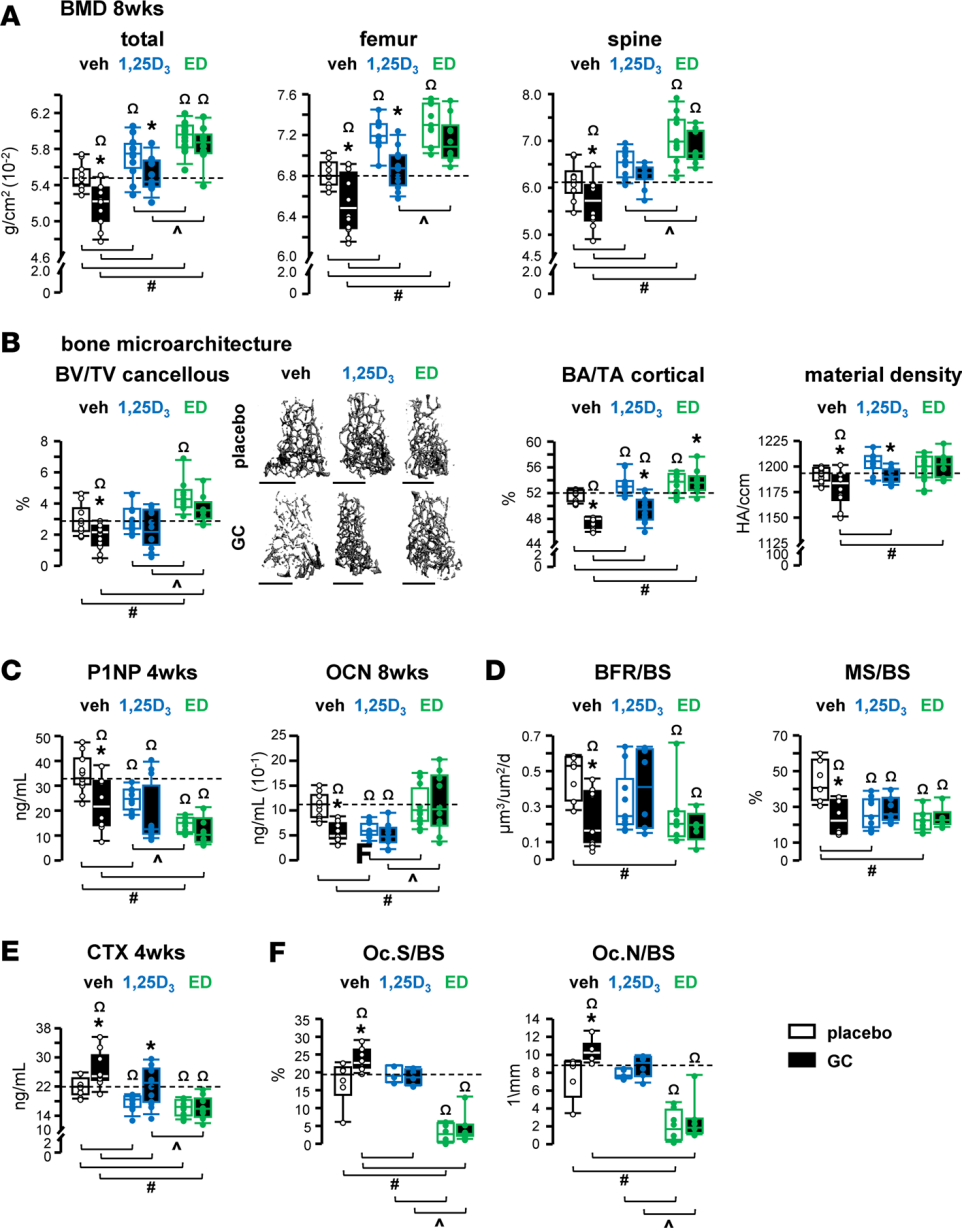
Increased VDR signaling protects the skeleton from bone loss and microarchitecture deterioration by blocking GC effects of remodeling. (**A**) BMD and (**B**) cancellous bone volume (BV) normalized by tissue volume (TV) and representative 3D reconstructed cancellous bone images. Scale bar: 1 mm. Cortical bone area (BA) normalized by tissue area (TA) and material density after 8 weeks of the indicated treatments: slow-release pellet implantation of either 2.1 mg/kg/d prednisolone or placebo and gavaging of 50 ng/kg/d 1,25D_3_, ED, or vehicle 5 times per week for 8 weeks. (**C** and **E**) Sera P1NP, OCN, and CTX levels. *n* = 10–12. (**D**) Histomorphometric quantification of bone formation rate (BFR) and mineralizing surface (MS) normalized by bone surface (BS) in longitudinal sections of lumbar vertebral L1–L3 cancellous bone. *n* = 5–10. (**F**) Osteoclast surface (Oc.S) and number (Oc.N) normalized to bone surface (BS). *n* = 5–8. *P < 0.05 vs. corresponding placebo treated, ^#^*P* < 0.05 vs. corresponding vehicle treated, ^*P* < 0.05 vs. corresponding 1,25D_3_ treated by 2-way ANOVA, Tukey’s post hoc test, ^Ω^*P* < 0.05 vs. placebo and vehicle-treated controls by 1-way ANOVA, Dunnett’s method post hoc test.

GC decreased total, femoral, and spinal bone mineral density (BMD) after 4 and 8 weeks (Figure 2A and Supplemental Figure 1A; supplemental material available online with this article; https://doi.org/10.1172/jci.insight.182664DS1) of administration compared with corresponding placebo groups, as previously reported (13, 14, 16, 25). Treatment with the VDR ligands 1,25D_3_ or ED increased BMD at all 3 sites regardless of GC presence. However, despite the skeletal gains with 1,25D_3_, GC exposure still blunted these benefits on total and femoral BMD, but not in the spine, suggesting a distinction in the cortical versus cancellous bone response to 1,25D_3_. Notably, 1,25D_3_ still increased BMD in GC-treated animals to the level exhibited by vehicle-placebo controls. In contrast, ED completely protected against GC-induced decreases in BMD at all 3 sites and time points. In addition, overall ED increased total and spinal BMD further than 1,25D_3_ in both placebo- and GC-treated animals. These findings support the notion that increased vitamin D signaling can block adverse, chronic GC effects on BMD, either partially with 1,25D_3_ or fully with ED.

GC exposure leads to a marked deterioration of skeletal microarchitecture in both cancellous and cortical bone (Figure 2B and Table 1). 1,25D_3_ fully prevented the deterioration of cancellous bone microarchitecture induced by GC, whereas ED only partially protected against the reduction in trabecular number or the increase in trabecular separation (Figure 2B and Table 1). In cortical bone, both 1,25D_3_ and ED partially prevented GC effects, as indicated by gains in bone area/tissue area (BA/TA), cortical thickness, and femoral material density accompanied by reductions in marrow area. ED further improved cancellous bone architecture with increases in bone volume/tissue volume (BV/TV) and trabecular thickness when compared with GC-treated controls, even to a higher extent than 1,25D_3_. In addition, ED, but not 1,25D_3_, prevented the reduction in femoral material density induced by GC.

The VDR ligands per se also improved cancellous and cortical bone microarchitecture (Figure 2B and Table 1). In cancellous bone, both metabolites increased trabecular thickness, and ED also increased BV/TV, and the effect of ED on BV/TV was to a higher extent than that of 1,25D_3_. Both metabolites additionally, increased cortical BA/TA and cortical thickness, and 1,25D_3_ also reduced the marrow area.

GC suppressed bone formation, as evidenced by reduced circulating levels of bone formation P1NP and OCN, lower bone formation rate/bone surface (BFR/BS), and mineralizing surface/BS quantified by dynamic histomorphometry (Figure 2, C and D). Both VDR ligands blocked GC-suppressive actions on bone formation indexes as detected in the circulation and at the tissue level. In addition, 1,25D_3_ by itself reduced P1NP and OCN, while ED decreased P1NP and BFR/BS, and both metabolites decreased mineralizing surface/BS. Mineral apposition rate (MAR) was not altered by any of the hormones in general, with an exception for a slightly lower MAR level with ED versus 1,25D_3_ within GC-treated animals (Supplemental Figure 1B).

GC stimulated resorption as CTX was elevated as early as 4 weeks and maintained by 8 weeks and by increased osteoclast surface and number on bone (Figure 2, E and F, and Supplemental Figure 1C). The GC-induced resorption occurred without alterations in circulating calcium or phosphate (Supplemental Table 1 and Supplemental Figure 1), with the exception of a transient increase of phosphate levels at 4 weeks that was not sustained. These findings are indicative of vitamin D–replete animals and also suggest an absence of pathologic impaired kidney/PTH/FGF-23 signaling at this stage of the GC disease. Both vitamin D metabolites protected against GC-induced increases in CTX, when compared with GC controls after 4 and 8 weeks. However, GC-induced increases were still detectable within 1,25D_3_-treated, but not ED-treated, animals, indicating a distinction in the level of protection between the 2 ligands (i.e., partial with 1,25D_3_ vs. full with ED). The partial 1,25D_3_ protection against CTX increases with GC also corresponds with the partial prevention of GC reductions in total and femoral BMD and the cortical bone fraction BA/TA (Figure 2, A and B). In contrast, 1,25D_3_ completely blocked GC increases in osteoclasts on cancellous bone (Figure 2F), which correspond to the full skeletal protection exhibited in BMD of the lumbar spine and the cancellous bone fraction BV/TV (Figure 2, A and B, and Supplemental Figure 1A). The protective actions of 1,25D_3_ also occurred without changes in circulating calcium and phosphate (Supplemental Table 1 and Supplemental Figure 1). For ED-treated animals, GC-induced resorption was fully blocked as indicated by CTX and osteoclast quantification (Figure 2, E and F), which also correspond to the full preservation of BMD at all 3 sites (Figure 2A and Supplemental Figure 1A). Additionally, ED alone profoundly decreased CTX measured at 4 and 8 weeks (Figure 2E and Supplemental Figure 1C) and osteoclast number (Figure 2F), whereas 1,25D_3_ decreased resorption transiently at 4 weeks. The marked ED suppression of resorption also occurred with a robust elevation of calcium levels detected at 4 and 8 weeks in all ED-treated animals, but overall, without alterations in phosphate levels (Supplemental Table 1 and Supplemental Figure 1). Taken together, the blockage of GC-stimulated resorption by both metabolites underlies the skeletal benefits exhibited on bone mineral and microarchitecture. No evident soft tissue calcifications were detected during dissections of muscle, liver, or heart. Furthermore, ex vivo dual-energy X-ray absorptiometry (DXA) scans of these tissues did not reliably detect calcifications, as exemplified for hearts in Supplemental Figure 4, with a femur included as a positive control.

### The VDR ligands partially protect against bone fragility by GC excess at the whole tissue and collagen fibril levels.

**Figure 3:**
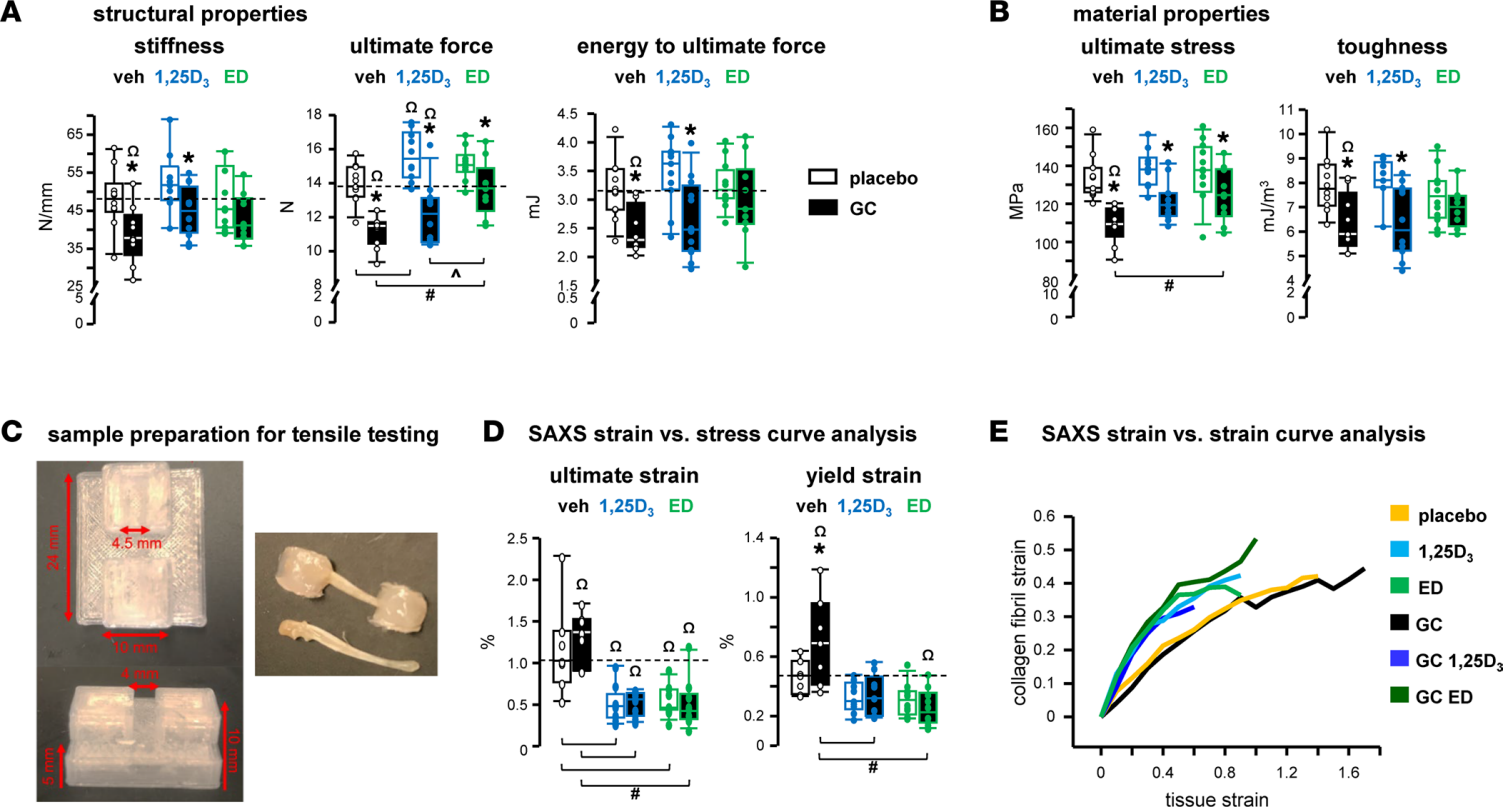
VDR ligands prevent GC adverse actions on bone mechanical properties, fully and partially, at the tissue and collagen fibril level, respectively. Mice were implanted with 2.1 mg/kg/d prednisolone or placebo slow-release pellets and gavaged with 50 ng/kg/d 1,25D_3_, ED, or vehicle 5 times per week for 8 weeks. (**A**) Structural and (**B**) material properties of bone, as assessed by 3-point bending of femurs. MPa, megapascal. *n* = 10–12. (**C**) Images for sample preparation for tensile testing utilized in synchrotron small-angle x-ray scattering (SAXS) analyses. (**D**) Ultimate and yield strain, as assessed by SAXS strain vs. stress curve analysis of combined ulnae and radii bones undergoing uniaxial tension testing. (**E**) Tissue strain was time matched to collagen strains (SAXS strain vs. strain curve analysis) at yield and maximum stress for comparisons of stress-carrying components with bone during deformation. *n* = 7–12. *P < 0.05 vs. corresponding placebo treated, ^#^*P* < 0.05 vs. corresponding vehicle treated, ^*P* < 0.05 vs. corresponding 1,25D_3_ treated by 2-way ANOVA, Tukey’s post hoc test, ^Ω^*P* < 0.05 vs. placebo and vehicle-treated controls by 1-way ANOVA, Dunnett’s method post hoc test.

As reported earlier (13, 16), GC weakened bone by impairing structural and material properties, quantified by femoral 3-point bending mechanical tests (Figure 3, A and B). Overall, 1,25D_3_ or ED alone did not change any of these indexes, but 1,25D_3_ significantly increased ultimate force. GC lowered all mechanical properties, even in 1,25D_3_-treated animals, and also decreased ultimate force and stress in ED-treated mice. However, bones from GC+1,25D_3_ and GC+ED mice exhibited similar mechanical properties to those of vehicle placebo control mice, indicating partial protection by the VDR ligands. In general, 1,25D_3_/ED exhibited similar effects on bone mechanical properties, except for an increase in ultimate force with ED- versus 1,25D_3_ in GC-treated mice.

Consistent with the increased resorption and decreased material density, GC negatively impacted the mineral/matrix interactions at the collagen fibril level, as assessed by tensile testing with small-angle x-ray scattering (SAXS) (Figure 3, C–E). GC increased both the ultimate and yield strain, indicating increased bone deformation (Figure 3D). In other words, bones from GC-treated animals lose their shape at lower applied forces. In contrast, both VDR ligands decreased ultimate and yield strain regardless of GC presence, thus bones maintain their shape at higher forces. These results indicate prevention of GC-induced deformation by VDR ligands, which is further supported by a shift induced by 1,25D_3_ and ED in the collagen fibril strain versus (bone) tissue strain curve analysis derived from SAXS, although the curves did not reach significance (Figure 3E and Supplemental Figure 2, A and B).

In summary, these findings detect GC-induced bone fragility in mechanical properties at the tissue and collagen fibril levels, which were, overall, prevented, partially at the tissue level and fully at the collagen fibril, by the VDR ligands.

### VDR ligands partially protect against skeletal muscle dysfunction induced by GC.

**Figure 4:**
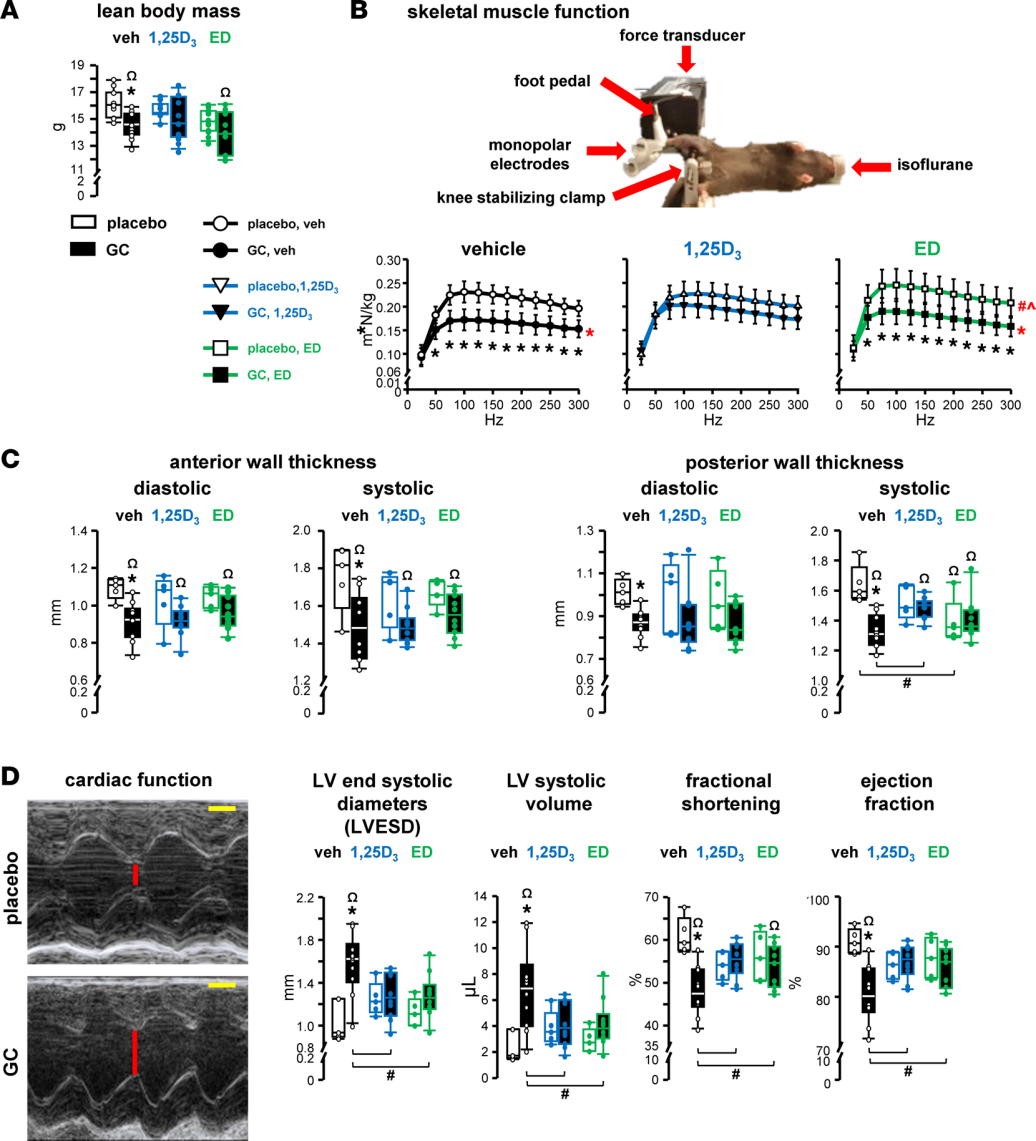
VDR ligands partially protect against skeletal and cardiac muscle dysfunction induced by GC. Mice were implanted with 2.1 mg/kg/d prednisolone or placebo slow-release pellets and gavaged 5 times per week with 50 ng/kg/d 1,25D_3_, ED, or vehicle for 8 weeks. (**A**) Lean body mass and (**B**) skeletal muscle function, as assessed by plantarflexion torque in vivo testing measured after 4 weeks of the indicated treatments. *n* = 10–12. *P < 0.05 vs. corresponding placebo treated, by 2-way ANOVA for **A** and by 2-way repeated-measures ANOVA, Tukey’s post hoc test for **B**. Main group effects are indicated by red symbols: red *P < 0.05 all corresponding placebos vs. all corresponding GC, red ^#^*P* < 0.05 all corresponding vehicles vs. all corresponding EDs, red ^*P* < 0.05 all corresponding 1,25D_3_s vs. all corresponding EDs by 2-way repeated-measures ANOVA, Tukey’s post hoc test. (**C**) Left ventricle (LV) wall thickness of the anterior and posterior surfaces at diastole and systole, as measured by Vevo2100 Imaging System ultrasound biomicroscopy system in vivo. (**D**) Representative images, LV end systolic diameters, LV systolic volume, fractional shortening, and ejection fraction generated from ultrasound echocardiograms (scale bars: 1 mm). *n* = 5 placebo-treated, *n* = 10–12 GC-treated. *P < 0.05 vs. corresponding placebo treated, ^#^*P* < 0.05 vs. corresponding vehicle treated, ^*P* < 0.05 vs. corresponding 1,25D_3_ treated by 2-way ANOVA, Tukey’s post hoc test, ^Ω^*P* < 0.05 vs. placebo and vehicle-treated controls by 1-way ANOVA, Dunnett’s method post hoc test.

GC decreased lean body mass, an index of skeletal muscle mass assessed by DXA, and impaired skeletal muscle function quantified in vivo by plantarflexion torque testing, which measures the posterior musculature compartment of the hindlimb as a functional unit (Figure 4, A and B). Both 1,25D_3_ and ED prevented reductions in lean body mass by GC. Furthermore, 1,25D_3_ fully prevented skeletal muscle weakness by GC. In contrast, ED failed to prevent GC-induced weakness. Neither VDR ligand by itself altered lean body mass, and 1,25D_3_ did not alter skeletal muscle strength. In contrast, ED overall increased muscle strength compared with vehicle-placebos and 1,25D_3_-placebos, as detected by main group comparisons for all frequency stimulations, although comparisons within each specified frequency did not reach statistical significance. In general, comparisons between the two metabolites revealed no detectable distinctions in lean body mass or skeletal muscle function, with the exception that ED elicited higher plantarflexion torque values than 1,25D_3_ when comparing all frequency stimulations as a main group effect.

The 1,25D_3_ preservation of skeletal muscle detected by lean body mass and function exhibited after 4 weeks (Figure 4, A and B) was not sustained, as GC decreased the wet weight of isolated muscles after 8 weeks (Supplemental Figure 1D). In addition, after 8 weeks, ED by itself notably lowered muscle wet weight, which was not further reduced by GC administration. Overall, 1,25D_3_ fully and ED partially protected against muscle wasting and weakness induced at early stages of GC excess.

This distinction between 1,25D_3_ and ED regarding muscle also corresponds with intrinsic differences between the metabolites regarding circulating calcium levels. By itself, ED, but not 1,25D_3_, induced increases in sera calcium after 4 weeks, which were sustained after 8 weeks of treatment (Supplemental Table 1 and Supplemental Figure 1). In contrast, calcium was not elevated in 1,25D_3_-treated animals, with the exception of a transient increase at 4 weeks with GC+1,25D_3_, which was not maintained at 8 weeks.

### VDR ligands preserve cardiac function against adverse GC actions in vivo.

GC decreased the wall thickness of the left ventricle (LV) at both the anterior and posterior surfaces at diastole and systole (Figure 4C), a recognized hallmark of early-phase eccentric hypertrophy induced by GC (17, 26). Both vitamin D_3_ metabolites prevented GC-induced LV thinning at both surfaces and contraction phases. However, the LV wall was thinner in all GC-treated mice compared with vehicle-placebo controls. Overall, the VDR ligands alone did not alter LV thickness, except for a slight decrease induced by ED in the posterior wall at systole (but not at any other surface or contraction phase). These findings demonstrate that both VDR ligands partially prevented GC-induced thinning of the LV wall.

At this early disease stage, GC did not alter LV mass index in absolute values or when normalized by each mouse’s own body weight or heart weight (Supplemental Figure 3, A and B). In addition, the VDR ligands did not alter LV mass or heart weight, and no differences in body weight were detected with GC or either VDR ligand alone or in combination. GC also induced early signs of cardiac dysfunction, as detected by increased LV end-systolic diameters and decreased fractional shortening, which drive increased LV systolic volume and decreased ejection fraction, respectively (Figure 4D). Although neither 1,25D_3_ nor ED alone affected these functional cardiac indexes, GC-induced cardiac dysfunction was fully prevented by 1,25D_3_ or ED.

### Targeting the atrogene pathway by pharmacologic and genetic means confers protection against disease by GC excess in the musculoskeletal and cardiac systems.

To investigate the impact of atrogene upregulation by GC to proteasomal activity, we examined whether proteasomal inhibition protects against GC action.

**Figure 5:**
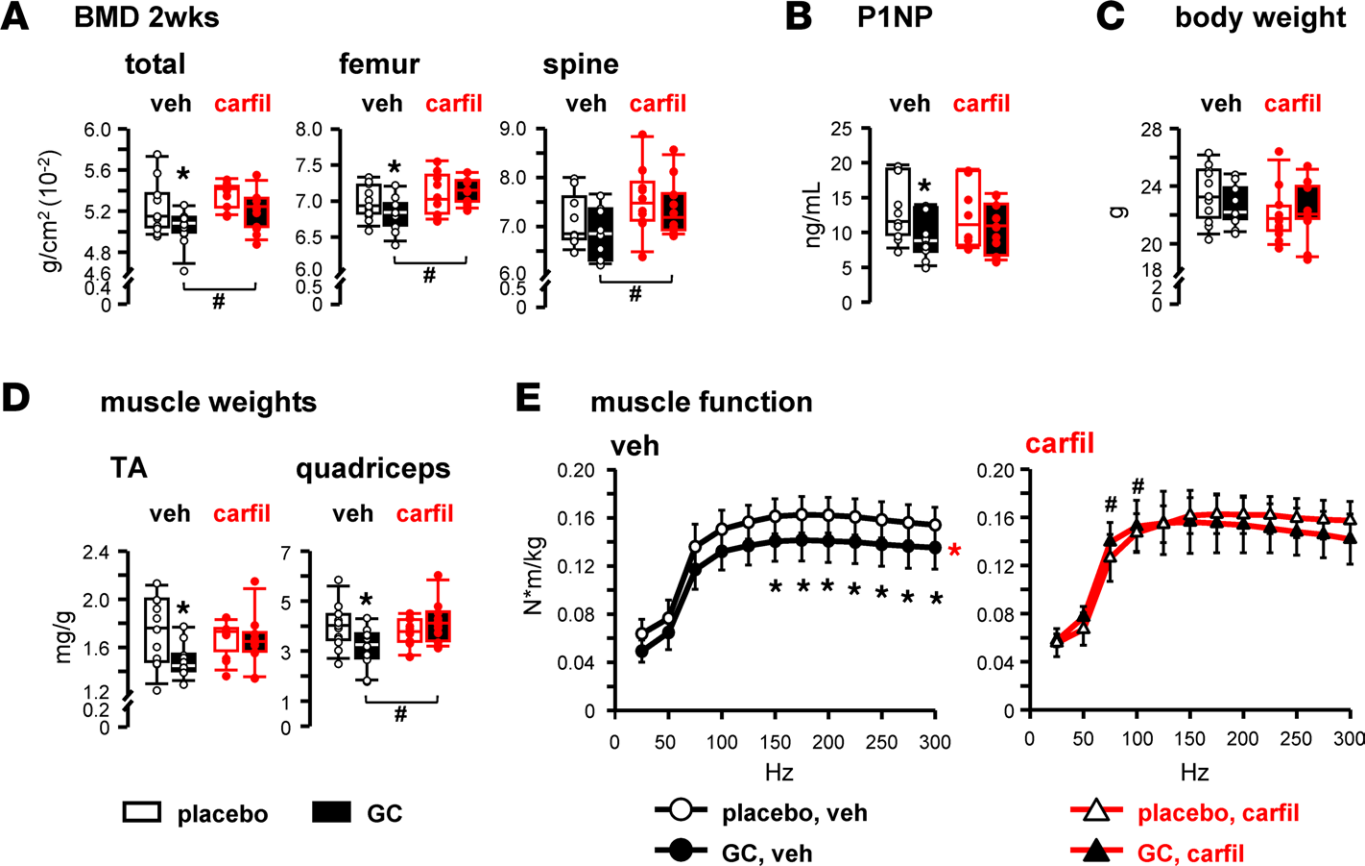
Interference with the atrogene pathway confers musculoskeletal protection against excessive GC. Mice were implanted with 2.1 mg/kg/d prednisolone or placebo slow-release pellets and treated with 5 mg/kg/d carfilzomib or vehicle by intraperitoneal injection 2 times per week for 2 weeks. (**A**) BMD, (**B**) sera P1NP, (**C**) body weights, and (**D**) isolated skeletal muscle weights 2 weeks after the indicated treatments. *n* = 10–12. *P < 0.05 vs. corresponding placebos, ^#^*P* < 0.05 vs. corresponding vehicle-treated, by 2-way ANOVA, Tukey’s post hoc test. (**E**) Skeletal muscle function, as assessed by plantarflexion torque in vivo testing measured after 2 weeks of the indicated treatments. *n* = 11–12. *P < 0.05 vs. corresponding placebos, ^#^*P* < 0.05 vs. corresponding vehicle-treated. Main group effects are indicated by red symbols: red *P < 0.05 all corresponding placebos vs. all corresponding GC, by 2-way repeated-measures ANOVA, Tukey’s post hoc test.

Induction of osteoblast apoptosis is a recognized feature of GC excess in vitro and in vivo (27, 28). We found that the proteasomal inhibitor bortezomib prevented dexamethasone-induced OB-6 osteoblastic cell death in vitro (Supplemental Figure 3C). Moreover, the decrease in BMD induced by GC was prevented at all 3 skeletal sites in vivo by the proteasomal inhibitor carfil (Figure 5A), which also prevented GC decrease in the bone formation marker P1NP (Figure 5B), without alterations in body weight (Figure 5C). Carfil also protected skeletal muscle from the adverse GC actions (Figure 5, D and E), preventing the decrease in wet muscle weight induced by GC. Carfil also abolished GC-induced muscle weakness detected at frequency stimulations of 150–300 Hz and increased muscle strength at 75–100 Hz. Carfil by itself did not affect BMD, markers of bone formation, muscle wet weight, body weight, or skeletal muscle function (Figure 5).

**Figure 6:**
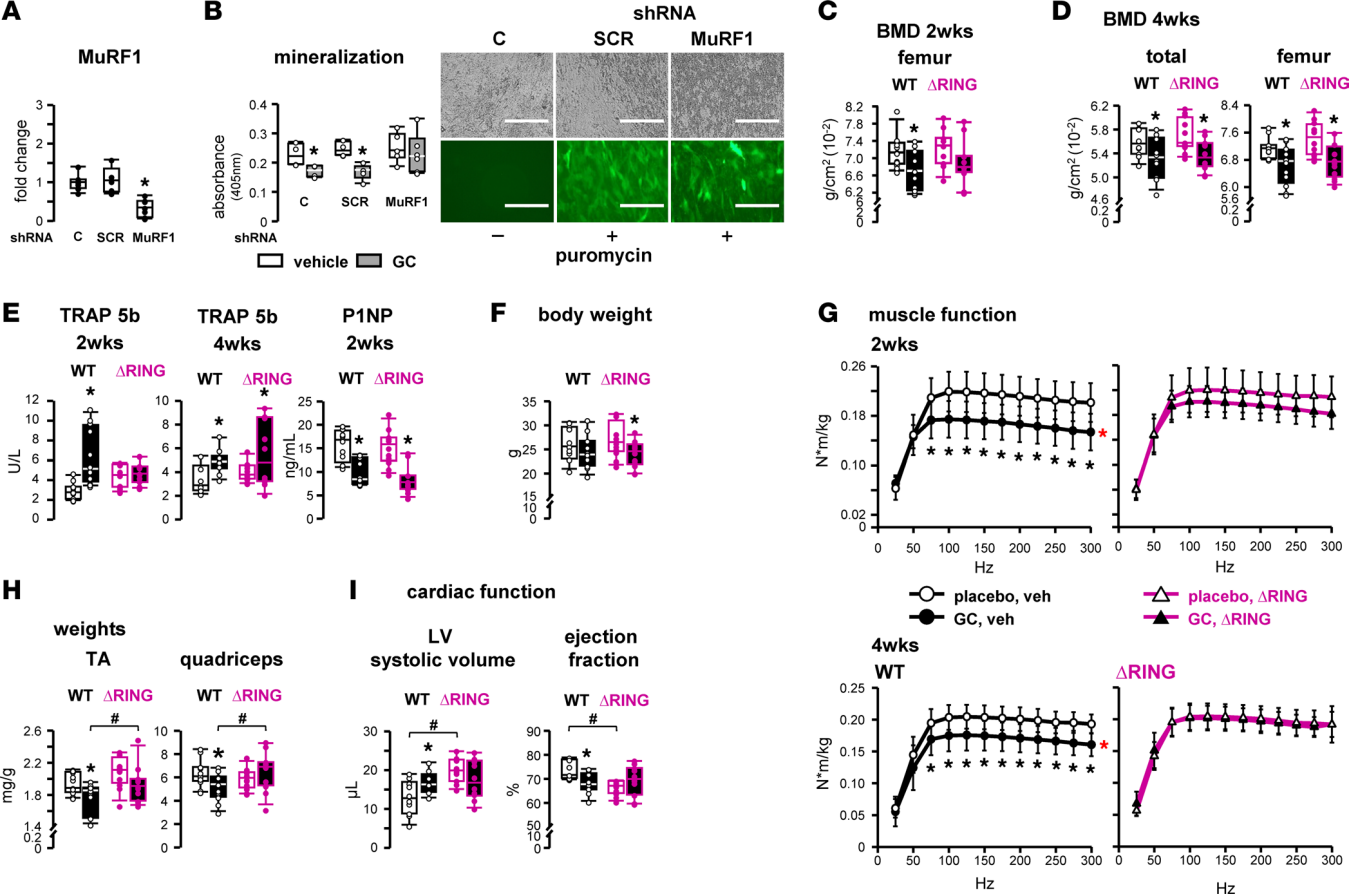
Loss of MuRF1-mediated ubiquitination prevents GC dysfunction in skeletal and cardiac muscle, but only temporarily in bone, in vivo. (**A** and **B**) *MuRF1* expression was quantified by qPCR in OB-6 cells not infected (controls [C]) or infected with GFP-labeled lentivirus containing scramble shRNA (SCR) or shRNA directed to silence *MuRF1*. (**A**) *P < 0.05 vs. non-infected cells by 1-way ANOVA, Dunnett’s method post hoc test. (**B**) Mineralization was visualized by Alizarin Red S staining followed by optical density quantification (absorption 405 nm), read in duplicate. Representative images for GFP visualization are shown. Scale bars: 200 μm. *n* = 4–6, *P < 0.05 vs. vehicle-treated, by Student’s *t* test. (**C**–**I**) WT littermates and mice lacking MuRF1-mediated ubiquitination due to deletion of the RING region (ΔRING) were implanted with slow-release pellets delivering 2.1 mg/kg/d (GC) prednisolone or placebo. (**C** and **D**) BMD and (**E**) sera TRAP 5b and P1NP, (**F**) mouse body weights, and (**H**) wet weight of isolated muscles. *n* = 10–12. *P < 0.05 vs. corresponding placebos, ^#^*P* < 0.05 vs. corresponding WTs, by 2-way ANOVA, Tukey’s post hoc test. (**G**) Skeletal muscle function, as assessed by plantarflexion torque in vivo testing, measured after 2 and 4 weeks of the indicated treatments. *n* = 10–12. *P < 0.05 vs. corresponding placebo treated. Main group effects are indicated by red symbols: red *P < 0.05 all corresponding placebos vs. all corresponding GC by 2-way repeated-measures ANOVA, Tukey’s post hoc test. (**I**) Left ventricle (LV) systolic volume and ejection fraction, as assessed by ultrasound echocardiography. *n* = 11–12. *P < 0.05 vs. corresponding placebos, ^#^*P* < 0.05 vs. corresponding WT, by 2-way ANOVA, Tukey’s post hoc test.

We next examined the specific role in GC action of the atrogene MuRF1. In vitro, GC failed to decrease matrix mineral production in *MuRF1* knocked down OB-6 cells whereas it reduced mineral deposition in noninfected control or scrambled control (SCR) cells (Figure 6, A and B). In vivo, genetically modified mice lacking the ubiquitination function of MuRF1 (ΔRING mice) (29) exhibited a partial and transient protection against GC, as the reductions in femoral and total BMD observed in WT littermate control mice were blunted in ΔRING mice at 2 but not at 4 weeks of GC treatment (Figure 6, C and D). Furthermore, the increase in circulating TRAP 5b induced by GC in WT mice was only corrected in ΔRING mice at 2 weeks but not at 4 weeks (Figure 6E). Moreover, GC decreased P1NP levels in both WT and ΔRING mice. Loss of MuRF1-mediated ubiquitination by itself did not alter BMD or markers of bone resorption or formation or body weight (Figure 6F). At 4 weeks, expression of the other atrogenes *Atrogin1* and *MUSA1* and of *UbC*, a polyubiquitin precursor crucial for proteosomal activity, is increased in bones of ΔRING mice (Supplemental Figure 3), suggesting that when the ΔRING mice lose protection from GC, there is a compensatory increase in other components of the proteosomal pathway.

In contrast to bone, skeletal and cardiac muscles were fully protected from GC-induced deterioration of tissue structure and function in ΔRING mice (Figure 6, G–I). Thus, in vivo muscle function was not impaired by GC in ΔRING mice (Figure 6G), and ΔRING mice were also protected from GC-induced muscle loss (Figure 6H). Similarly, whereas WT littermates exhibited GC-induced cardiac dysfunction, ΔRING mice were protected. Furthermore, GC increased LV systolic volume and LV end-systolic diameters and decreased ejection fraction and fractional shortening in WT but not in ΔRING mice (Figure 6I and Table 2). The lack of GC effects in LV wall thickness, LV mass, or body weight indicates an earlier disease state in the 4-week versus 8-week study (Figure 4, C and D). Of note, ΔRING mice administered placebo displayed a basal phenotype characterized by signs of inefficient cardiac contraction/function and increased heart weight compared with WT littermate mice. These findings suggest that the cardiac disease exhibited by mice with global *MuRF1* deletion (30) is due to the MuRF1-mediated ubiquitination versus other functions.

## Discussion

**Figure 7:**
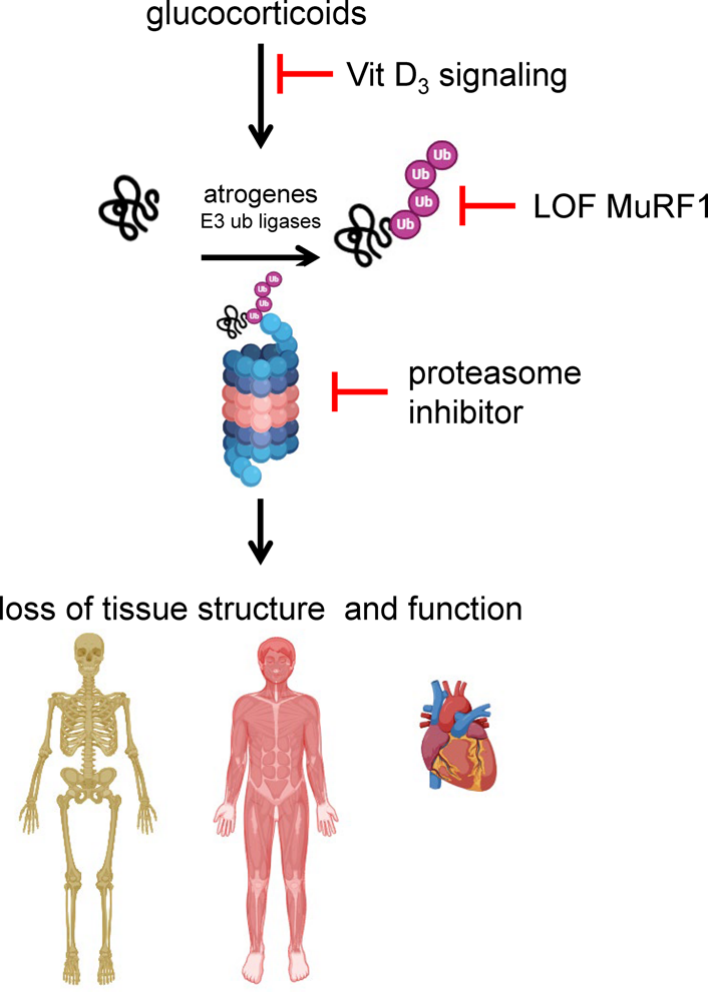
Atrogene upregulation is a central mechanistic hub underlying the damaging actions of GC excess in bone, skeletal muscle, and the heart. Interfering with the E3 ubiquitin (ub) ligase (atrogene) pathway via increased vitamin D_3_ (Vit D_3_) signaling blocks the deterioration of tissue structure and function in the musculoskeletal and cardiac systems. Likewise, usage of proteasomal inhibitor carfilzomib preserves bone and skeletal muscle in the setting of excessive GC, indicating that proteasomal-driven protein catabolism mediates musculoskeletal atrophy by GC. Likewise, genetic loss of function of MuRF1-mediated ubiquitination protects against adverse GC actions in muscle tissues (both skeletal and cardiac) and initially protects bone. Overall, these in vivo findings demonstrate (a) that the atrogene pathway is commonly upregulated in excessive GC disease in 3 distinct and highly specialized tissues, bone, skeletal muscle, and the heart; (b) that increased vitamin D_3_ signaling preserves tissue structure and function by interfering with GC actions on the atrogene pathway in each of these organs; and (c) that MuRF1’s molecular ubiquitination function is the mechanistic contributor to the loss of tissue structure and function in skeletal and cardiac muscle tissues.

This study demonstrates that upregulation of atrogene expression underlies the damaging actions of GC in the bone, skeletal muscle, and the heart and that pharmacologic or genetic interference with the atrogene pathway prevents the mass/structure and function deterioration induced by GC in these tissues (Figure 7). Our work identifies the atrogene MuRF1 and its ubiquitination function as a critical mediator of GC action. We also show that two pharmacologic strategies targeting proteasomal proteolysis protect against GC actions: ligands of the vitamin D receptor (VDR) that prevent atrogene upregulation by GC and direct inhibition of the proteasome. These findings pave the way toward the development of strategies protecting against undesirable and life-threatening GC side effects in multiple tissues by targeting a single pathway. Thus, our findings challenge the standard of care in which GC-induced pathologies are treated separately in bone, muscle, and the heart.

GC-induced bone disease is treated with antiresorptive agents (bisphosphonates as alendronate, risedronate, zolendronic acid, or anti-RANKL antibody denosumab), with pure anabolic agents (teriparatide or abaloparatide), or with the dual-action anabolic/antiresorptive agent antisclerostin antibody (romosozumab), all of which lower the fracture risk (31–34). Yet, these strategies exhibit side effects. Inhibition of resorption stops GC-induced bone loss but suppresses bone formation even more (33, 35) and further reduces bone turnover over GC alone, resulting in microdamage accumulation, avascular osteonecrosis, and/or atypical low-trauma fractures (36, 37). Anabolic therapies are only effective for a limited period of time, as peak levels of bone formation are not sustained and eventually decrease (33, 34, 38–40). The effect of the antisclerostin antibody decreases over time as well, and this therapy is only approved by the FDA for high fracture risk osteoporotic women due to potential adverse cardiovascular risks (39, 41). All these treatments have substantial costs, and there is limited health insurance coverage (42, 43). Bone and muscle function as a mechanical unit; however, antiosteoporotic therapies do not protect from skeletal muscle atrophy. Furthermore, there are no effective approved therapies for sarcopenia. Regarding GC-associated CVD, it is recommended the use of the lowest dose for the shortest duration of synthetic GC with low affinity for the mineralocorticoid/aldosterone receptor (MR) (44). In addition, MR antagonists use and close monitoring for heart failure are recommended (45, 46). However, MR antagonists induce kidney dysfunction and do not protect from GC receptor–induced atrioventricular block (47). Overall, there is a clear need for the development of new strategies to mitigate the damaging and potentially fatal side effects of GC in multiple tissues.

Our study shows that activation of VDR signaling with the active metabolites 1,25D_3_ or ED offsets the actions of GC in the musculoskeletal and cardiac systems. GC therapy increases the risk of falls by 2.8-fold within the first 3 months of treatment (48), with concomitant enhanced prevalence of bone fractures (28, 49). In contrast, vitamin D supplementation improves muscle function to reduce falls and lowers bone fracture risks in some studies (23, 50–55). However, the decrease in falls and/or the gain in muscle strength associated with vitamin D supplementation are not consistently detected (23, 56–59). The discrepancies in observed benefits in muscle and bone of vitamin D supplementation have been attributed to variations in individual baseline vitamin D status (replete, insufficient, deficient), dosage (200–200,000 IU), frequency (daily, monthly, quarterly), and duration (6 months to 5 years) of treatments; presence of calcium supplementation; levels of total versus free, nonprotein bound, 25OHD_3_; and fracture type (hip, vertebral, nonvertebral, fall related) (59–61). Nevertheless, despite the clinical inconsistencies, our findings show that increased VDR activation lessens musculoskeletal atrophy in the context of GC excess and vitamin D replete status.

Although both VDR ligands largely exhibited similar benefits, our study detected notable distinctions. We originally selected ED based on previous reports showing it to be less hypercalcemic than 1,25D_3_ in rodent models (62). However, we found that ED induced hypercalcemia earlier and to a higher extent compared with 1,25D_3_ (Supplemental Table 1 and Supplemental Figure 1). Consistent with our findings, increased circulating calcium was reported in estrogen-deficient rodents treated with ED (62–64). Furthermore, clinical reports detected hypercalcemia as the most common adverse reaction to ED administration; although this side effect only occurs in 0.88% of the participants, with most of these individuals also exhibiting renal impairment (65). Therefore, less hypercalcemic VDR ligands might represent alternative strategies to prevent GC-induced musculoskeletal and cardiac disease.

Another difference between the VDR ligands is the superior benefits of ED compared with 1,25D_3_ at all 3 bone sites, which correspond to stronger suppression of bone resorption. In line with our findings, ED lowered osteoclasts in other rodent studies (63, 64) and decreased resorption markers in clinical studies (65). Only 1,25D_3_ exhibited protective effects on GC-induced muscle weakness. ED’s inability to prevent muscle weakness might be due to its earlier and higher hypercalcemic action, which causes muscle fatigue and weakness (66–68). Indeed, mice treated with ED alone exhibited lower tibialis anterior and quadriceps muscles weight, potentially resulting from ED-induced hypercalcemic muscle loss. Evidently, this hypercalcemic effect of ED masks any potential protective effect of the ligand, whereas the beneficial effect of 1,25D_3_ is patent because of its lower hypercalcemic action. These findings suggest that VDR activation exerts in skeletal muscle two distinct actions: a direct protective effect due to interference with the atrogene pathway and a secondary adverse muscle wasting action mediated by increased calcium absorption/reabsorption in the intestine/kidney leading to hypercalcemia. Additionally, the distinct bone and muscle responses to the VDR ligands might be explained by different pharmacokinetics and/or binding affinity for the vitamin D binding protein or the VDR (69–71). ED has a notably longer systemic half-life in circulation compared with 1,25D_3_, potentially explaining its greater potency (69, 71, 72). Nonetheless, our study demonstrates a clear proof of concept that pharmacologic VDR activation prevents GC action in bone and skeletal muscle. Future studies are needed to validate the notion that the vitamin D analogs act directly through the VDR expressed in muscle.

Our study revealed that the VDR ligands exhibit superior skeletal protection compared with either pharmacologic or genetic inhibition of proteasomal proteolysis, whereas both interventions were equality effective in protecting skeletal muscle and the heart from GC excess. While both 1,25D_3_ and proteasomal inhibitors are clinically available treatments, 1,25D_3_ might be the safer strategy as proteasomal inhibitors are linked to increased cardiovascular toxicity in patients with active multiple myeloma disease receiving also a combination of anticancer therapies (73).

Importantly, our study demonstrates protection from GC excess by 1,25D_3_ or ED in vitamin D–replete status, as mice were fed a regular diet, exhibited no changes in circulating calcium, and displayed only a small and transient increase in circulating phosphate after 4 but not 8 weeks of GC treatment (Supplemental Table 1 and Supplemental Figure 1). These preclinical findings support the notion that VDR activation might be beneficial in the context of GC excess, even in vitamin D_3_–sufficient patients, in addition to insufficient or deficient individuals. This concept is consistent with the current guidelines of the American College of Rheumatology that recommend vitamin D supplementation in GC-induced osteoporosis without discriminating vitamin D status (74). Nevertheless, future studies are warranted to provide the mechanistic basis of the interplay between vitamin D and GC in the musculoskeletal system.

Regarding the cardiovascular system, GC excess increases the risk for cardiovascular events and promotes heart failure (11, 75, 76). Remarkably, low circulating 25OHD_3_ is associated with increased risk for CVD, CVD-related mortality, coronary heart disease, peripheral arterial disease, and heart failure, as detected in a meta-analysis involving over 65,000 participants and reported by the National Health and Nutrition Examination Survey (77–80). Some clinical reports, but not all (59, 81), have also recently detected improved survival outcomes and additional cardiac/cardiovascular benefits with vitamin D supplementation in patients with heart failure (24, 82) or VDR agonists in patients with chronic renal failure (83, 84). Overall, these clinical reports and our findings support the notion that increased vitamin D signaling could aid in other cardiac/cardiovascular pathologies, like the one induced by GC excess. However, future studies are warranted to determine the dose and frequency/route of administration of VDR ligands that elicit VDR activation benefits without inducing hypercalcemia.

The studies reported here are the first to our knowledge to link VDR activation with atrogene expression in the context of GC excess in bone, muscle, and the heart. Interestingly, VDR ligands downregulated both *MuRF1* and *Atrogin1* similarly in bone and muscle, but only *MuRF1* in the heart, readily corresponding to improving cardiac function. We also identified protein ubiquitination as the critical molecular function of MuRF1 required for GC damaging actions in these tissues, as mice lacking the RING domain in MuRF1 are protected from GC action. However, bone is only temporary protected, suggesting that other long-term GC-induced effects cause bone loss even in the absence of MuRF1-mediated protein ubiquitination/degradation. MuRF1 has multiple molecular functions in skeletal and cardiac muscle, as it is a scaffold for focal adhesion kinases; binds serum release factor (85), titin (86), and RACK1 (87); and can induce transcription via translocation into the nucleus (86). Future studies are warranted to ascertain whether MuRF1 exhibits these additional molecular functions in bone and whether alternative MuRF1 roles contribute to GC-induced osteoporosis.

In summary, this study demonstrates that the MuRF1/atrogene pathway underlies GC action in bone, muscle, and the heart, and it can be pharmacologically or genetically targeted to confer protection against the damaging actions of GC in the 3 tissues.

## Methods

### Sex as a biological variable.

Bone responses to GC excess are similar for male (88–90) and female (14, 16, 25) mice. In addition, male mice caged together are prone to fight; and sexual dimorphic responses to vitamin D metabolites or proteasomal inhibitors have not been reported. Therefore, we selected female C57BL/6J mice for the pharmacologic intervention studies utilizing ligands 1,25D_3_, ED, or carfil. For the ΔRING (29) and WT littermate studies, female and male mice were equally utilized to account for the possibility of sex being a biological variable.

### Mice.

Mice were fed a regular diet (Teklad Global 18% Protein Extruded Rodent Diet Sterilizable, 2018SX, Harlan/ENVIGO), received water ad libitum, and were maintained on a 12-hour-light/dark cycle in polycarbonate cages. Mice were implanted with 90-day slow-release pellets delivering placebo or 2.1 mg/kg/d (GC) prednisolone (Innovative Research of America) (13, 14, 16, 25). GC treatment did not affect the body weight (Figure 5 and Supplemental Figure 3B). Three days before pellet implantation, C57BL/6J mice were gavaged with vehicle (Medium-chain triglyceride, Amazon), 1,25D_3_ (Santa Cruz), or the active vitamin D_3_ derivative eldecalcitol-71 (ED) (63, 91) (BOC Sciences) 5 times per week at 50 ng/kg/d for 8 weeks. For the proteasomal inhibitor experiment, 3 days before pellet implantation, C57BL/6J mice were treated with carfil (Fisher Scientific) 5 mg/kg/d or vehicle (10% captisol in 10 mM citrate solution) i.p. 2 times per week for 2 weeks and then euthanized. All mice were injected 10 and 3 days prior to sacrifice with 0.6% calcein (30 mg/kg; Sigma-Aldrich) and 1.0% alizarin red (50 mg/kg; Sigma-Aldrich) solutions, respectively. Mice were euthanized by 2% isoflurane (Abbott Laboratories) with a Drager 19.1 anesthetic Vaporizer and then by cervical dislocation. Hindlimb muscles and hearts were then isolated and weighed. To genotype ΔRING mice, genomic DNA was extracted from tail/ear, followed by PCR reaction using the following primers: ΔRING forward primer, GCCCAGACTTTGGGAGGAG, and reverse primer, GCACGCAGCCTCTGAGATG, with probe FAM–TGCTGTGACCATGTTCTTCTCGCCA–TAMRA.

### BMD.

Lean body mass and BMD of the total body, excluding head and tail, the lumbar spine (L1-L6), and the femur were measured by DXA by using a PIXImus II densitometer (GE Medical Systems, Lunar Division) (13, 14) in mice anesthetized with isoflurane. DXA was performed 2–4 days before (initial) administration of any treatments and 1-day prior to euthanasia (final). Mice were randomized to the experimental groups based on initial BMD values. Whole hearts were ex vivo imaged (Faxitron UltraFocus, Hologics) and measured by DXA (PIXImus II densitometer, GE Medical Systems, Lunar Division). Hearts from mice exposed to the same experimental condition were scanned together along with a mouse femur to validate the approach and control reproducibility and variability of the DXA scan.

### Muscle function testing.

In vivo muscle function was quantified using the 1205A Whole Mouse/Rat Test System (Aurora Scientific Inc.) as described previously (14). Briefly, mice were anesthetized with isoflurane and placed in the supine position with the right ankle at 90 degrees of dorsiflexion and the leg perpendicular to the foot pedal. Two sterile monopolar stimulated electrodes were inserted subcutaneously near the tibial nerve. Electrode placement and stimulation current were adjusted to achieve the maximum twitch response and then increased to approximately 35 mA for plantarflexion to ensure supramaximal stimulation of the muscle fibers. The maximum isometric torque (N*m) was recorded for 25–300 Hz stimulation frequencies, with a pulse width of 0.2 milliseconds and train duration of 200 milliseconds, and then normalized by mouse body weight (kg). Data were recorded using the Dynamic Muscle Control/Data Acquisition and Dynamic Muscle Control Data Analysis programs (Aurora Scientific Inc.).

### Echocardiography.

Echocardiography was performed by collecting short-axis B mode recordings using a Vevo2100 Imaging System (VisualSonics) ultrasound biomicroscopy system with at least 3 independent waveforms per image for all LV data, as described previously (17, 85).

### Mechanical testing.

The mechanical properties of femoral mid-diaphysis were assessed by 3-point bending using standard methods (16, 92, 93). Femurs were placed with the posterior side down on the bottom support (9 mm wide) with the descending probe contacted with the central anterior surface and loaded at a rate of 2 mm/min until failure (100P225 Modular Test Machine), as described previously (16, 93). Structural/extrinsic properties were derived from the load/displacement curves and then normalized by the femoral geometry and volume quantified by micro-CT to calculate the material/intrinsic properties, following published equations (16, 92–94).

### Bone microarchitecture.

Femurs were dissected, cleaned of soft tissue, and stored in saline-soaked gauze at –20°C until micro-CT scanning at 10 μm resolution (ScancoMedical, μCT35). Cancellous bone of the distal femur and cortical bone of the femoral midshaft were quantified as previously published (16) following standard nomenclature (95). Representative 3D reconstructed images of bones having numerical BV/TV values closest to the average per experimental condition were chosen.

### Serum biochemistry.

Sera were from blood collected after 3 hours of fasting and within 24 hours of the last treatment by venipuncture of the facial vein with a sterile 18-gauge needle. N-terminal propeptide of type I procollagen (P1NP), C-terminal telopeptides of type I collagen (CTX), and tartrate-resistant acid phosphatase form 5b (TRAP 5b) were measured using enzyme-linked immunosorbent assays (Immunodiagnostic Systems Inc.) (13). Osteocalcin (OCN) was measured using the Mouse Osteocalcin KIA Kit (Alfa Aesar) (16).

### Bone histomorphometry.

Lumbar vertebrae (L1–L3) fixed in 10% neutral buffered formalin were embedded undecalcified in methyl methacrylate, as described previously (13, 16). Dynamic histomorphometry was performed in 7 μm unstained bone sections under epifluorescence microscopy. Histomorphometric analysis was performed with a computer and digitizer tablet (OsteoMetrics) interfaced to an Olympus BX51 fluorescence microscope (Olympus America Inc.). Osteoclasts were quantified on L2 thin sections stained for TRAP and counterstained with Toluidine Blue (Sigma-Aldrich), as previously published (13, 16).

### SAXS.

Ulnae and radii were cleaned of soft tissue and stored in Hank’s Balanced Salt Solution (Gibco) soaked gauze at –20°C until SAXS testing was performed, as described previously (96). Briefly, synchrotron SAXS assessed the collagen fibril deformation during uniaxial tension testing of combined ulnae and radii bones at a beamline 7.3.3. at the Advanced Light Source (LBNL), in situ with a TST350 Tensile Testing Stage (Linkam Scientific Inc.) at a displacement rate of 2.5 μm/s and exposed to x-rays of 10 keV for 0.1 seconds every 5 seconds (97). Tissue strain was time matched to collagen strains at yield and max stress for comparisons of stress-carrying components with bone during deformation.

### Cells and apoptosis quantification.

Murine bone marrow–derived OB-6 osteoblastic cells were cultured as described previously (98). Cells were plated at a density of 15,000 cells/cm^2^ and cultured overnight with MEMα, 2% FBS, and 1% penicillin/streptomycin (Gibco). Cells were then treated with 3 nM bortezomib or vehicle (DMSO) for 1 hour, followed by 1 μM dexamethasone or vehicle (EtOH) for 24, 48, or 72 hours. Trypan Blue (Sigma-Aldrich) uptake was utilized to assess cell death, as previously published (25). Results are reported as percentage of dead cells normalized by the total cell number.

### MuRF1 knockdown.

Four different silencing pGFP-C-shLenti vectors directed to *MuRF1* and 1 SCR were designed (OriGene). OB-6 cells were cultured to 85%–90% confluence in MEMα**,** 10% FBS, and 1% penicillin/streptomycin, followed by infection with lentiviral particles at a multiplicity of infection of 10 and overnight incubation with 8 μg/mL polybrene (Sigma-Aldrich). The next morning additional growth medium containing 20% FBS was added without removing viral particles. Forty-eight hours after infection, medium was replaced with 2.5 μg/mL puromycin (Gibco) containing 20% FBS medium for 3 days. Transduced GFP-positive OB-6 cells were then cultured and maintained in 1 μg/mL puromycin.

### Mineralization assay.

OB-6 cells were plated at 5,000 cells/cm^2^ in MEMα containing 10% FBS and 1% penicillin/streptomycin, as described previously (25). At confluence, medium was replaced with osteogenic medium consisting of 50 μg/mL ascorbic acid and 10 mM β-glycerophosphate with 1 μM dexamethasone or vehicle (EtOH) with or without 3 nM bortezomib or vehicle (DMSO) for 6 or 10 days. Every 2–3 days, half of the osteogenic medium was replaced. Matrix mineral production was visualized by Alizarin Red S staining, followed by microplate reader quantification at absorbance 405 nm.

### RNA extraction and qPCR.

TRIzol (Invitrogen) was used for total RNA extraction, and cDNA was synthesized using the high-capacity cDNA reverse transcription kit (Applied Biosystems), as described previously (13, 14). Primers and probes for qPCR were designed using the Assay Design Center (Roche Applied Science) or were commercially available (Applied Biosystems). Relative mRNA expression levels were normalized to the housekeeping gene ribosomal protein, large P2 (*Rplp2*) or *Gapdh* by using the 2 to the power of negative ΔCt method as previously published (3, 5).

### Ex vivo cultures.

Bones and skeletal muscles were harvested from C57BL/6J mice and maintained in medium (MEMα for bones and DMEM for muscles) containing 10% FBS and 1% penicillin/streptomycin overnight (14). Cultured tissues were treated with 1 μM dexamethasone or vehicle (EtOH) with or without 1,25D_3_ (10 nM) or vehicle (EtOH) for 6 hours, and then mRNA was isolated as described previously (14).

### Statistics.

Data are expressed as box plots with overlaid dot plots, where each dot represents an individual mouse/sample, and the median is indicated by a line mid-box. Sample differences were assessed using SigmaPlot 14.5 (Inpixon), with the appropriate analysis indicated in corresponding legends. Means of experimental groups were detected as different by 1- or 2-way ANOVA. For skeletal muscle function in vivo assessment, maximum plantarflexion torque values were normalized by each mouse’s own body weight and analyzed using a mixed-model, 2-way repeated-measures ANOVA, as previously published (14). All pairwise multiple comparison procedures within 1-way or 2-way ANOVAs were followed by pairwise comparisons by Tukey’s or Dunnett’s method post hoc tests. *P* values of less than 0.05 were considered significant.

### Study approval.

Animal procedures were approved by the Institutional Animal Care and Use Committee of Indiana University School of Medicine or the Division of Laboratory Animal Medicine of the University of Arkansas for Medical Sciences. Animal care was carried out in accordance with institutional guidelines.

### Data availability.

All manuscript data sets are provided in the Supporting Data Values file.

## Author contributions

TB designed the research. AYS, MC, KM, CAS, ES, JS, PV, BA, and MSW performed the research. AYS, CAS, BA, and MSW quantified the data. AYS, CAS, MSW, MB, TA, and TB analyzed and interpreted the data. AYS and TB wrote the paper with edits/comments from CAS, TA, and MB.

## Supplementary Material

Supplemental data

Supporting data values

## Figures and Tables

**Table 1:**
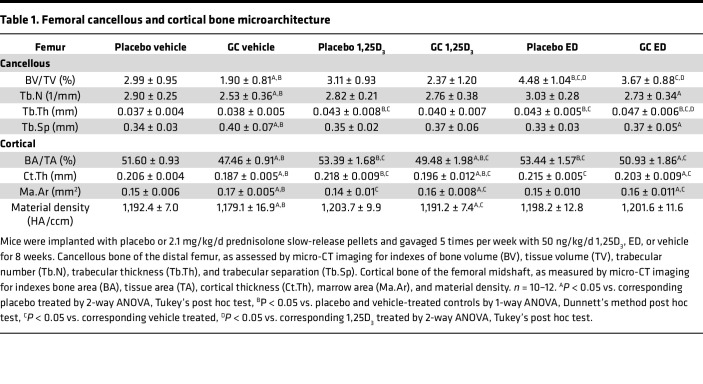
Femoral cancellous and cortical bone microarchitecture

**Table 2:**
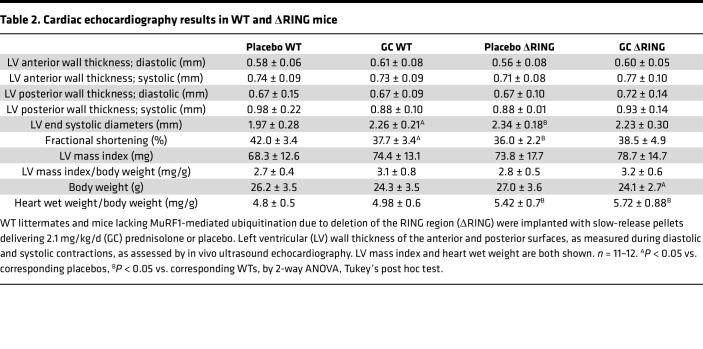
Cardiac echocardiography results in WT and ΔRING mice

## Version 1 10/15/2024

In-Press Preview

## Version 2 11/08/2024

Electronic publication
