# Supplementary material for: Pharmacologic or genetic interference with atrogene signaling protects against glucocorticoid-induced musculoskeletal and cardiac disease
Source: JCI Insight. 2024 Nov 8;9(21):e182664. doi: 10.1172/jci.insight.182664 (PMC11601705; doi:10.1172/jci.insight.182664)
Supplement: Supplemental data [file jciinsight-9-182664-s102.pdf]

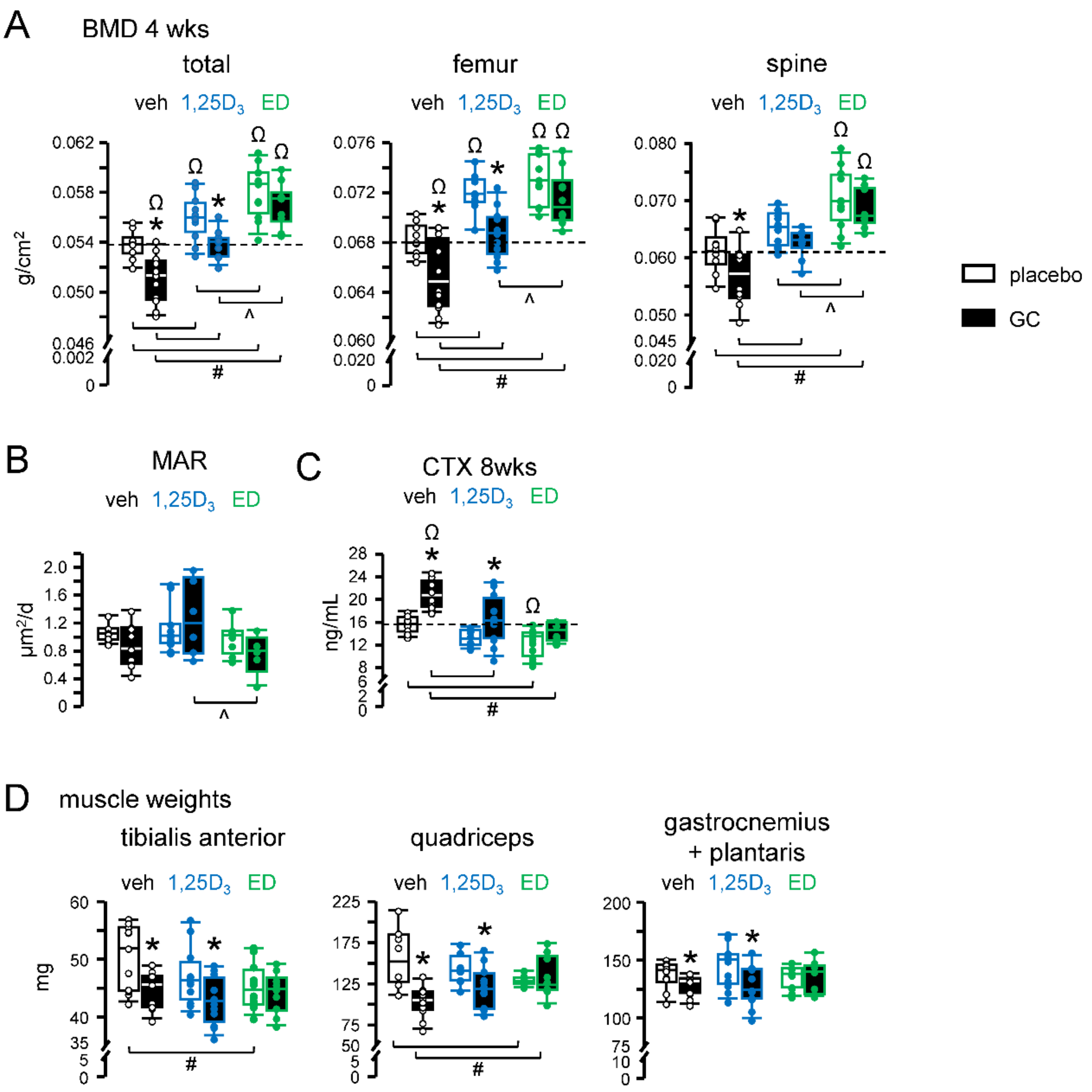

Supplemental Figure 1. GC and VDR metabolite actions upon the musculoskeletal tissues. Mice were implanted with 2.1 mg/kg/d prednisolone or placebo slow-release pellets and gavaged 5x/wk with 50 ng/kg/d $1,25D_3$ or ED or vehicle for 8 weeks. (*A*) BMD, (*C*) sera CTX, and isolated (*D*) wet weights of the indicated hindlimb muscles. n=10-12. (*B*) Mineralizing apposition rate (MAR) quantified by dynamic histomorphometry in longitudinal sections of lumbar vertebral L1-L3 cancellous bone. n=5-10 *P<0.05 vs. corresponding placebos, #P<0.05 vs. corresponding vehicle-treated, ^P<0.05 vs. corresponding $1,25D_3$-treated, by two-way ANOVA, Tukey post hoc test. ΩP<0.05 vs. placebo and vehicle-treated controls by one-way ANOVA, Dunnett's Method post hoc test.

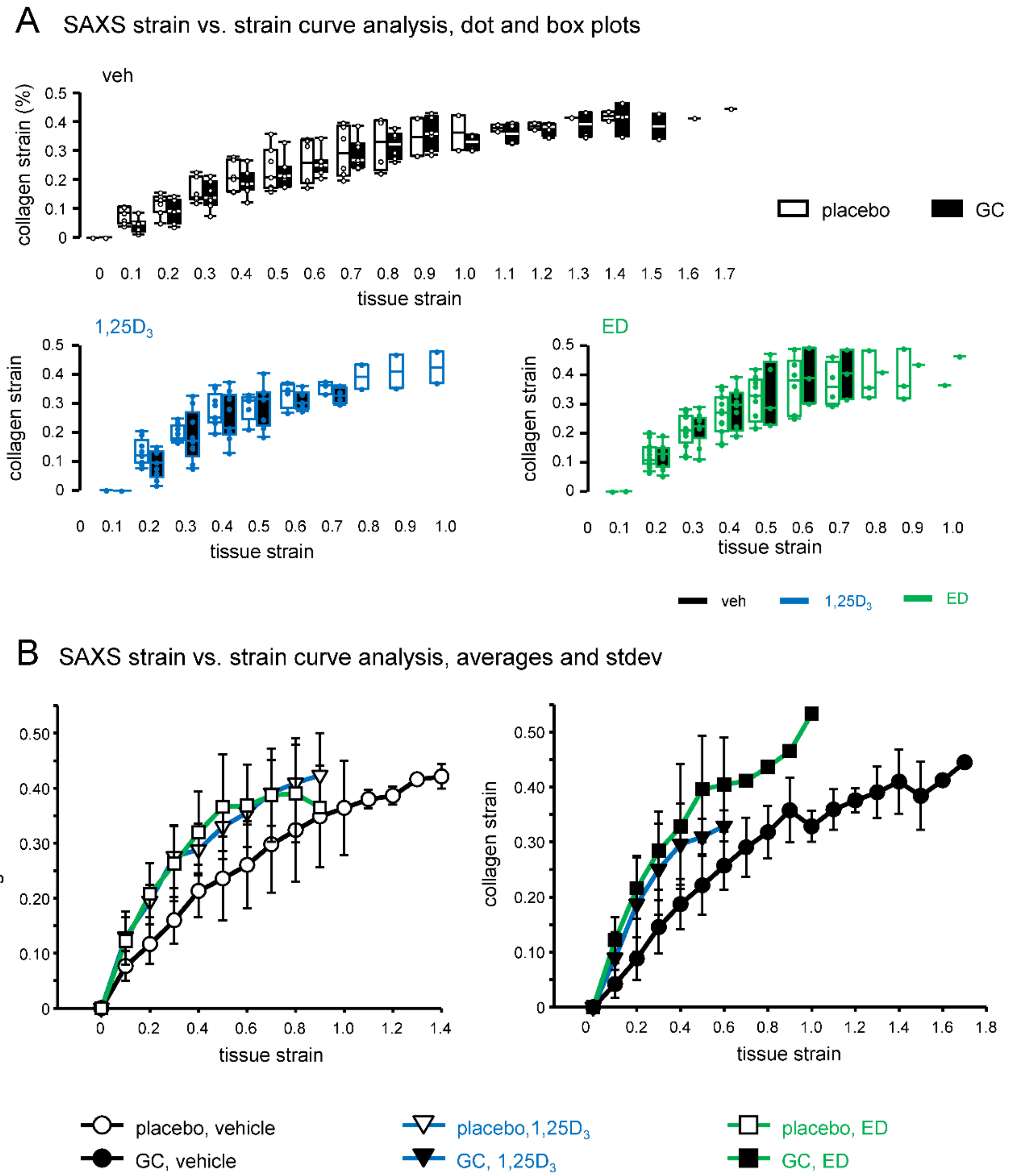

Supplemental Figure 2. Dot and box plot graphical representation of strain vs. strain synchrotron small angle x-ray scattering (SAXS) data. Mice were implanted with 2.1 mg/kg/d prednisolone or placebo slow-release pellets and gavaged 5x/wk with 50 ng/kg/d 1,25D3 or ED or vehicle for 8 weeks. Tissue strain was time-matched to collagen strains (strain vs. strain curve analysis) at yield and maximum stress for comparisons of stress-carrying components with bone during deformation. (*A*) Dot and box plots indicating each individual data point and (*B*) line graphs

indicating the average and standard deviation values for findings generated by SAXS strain vs. strain curve analyses. n=7-12.

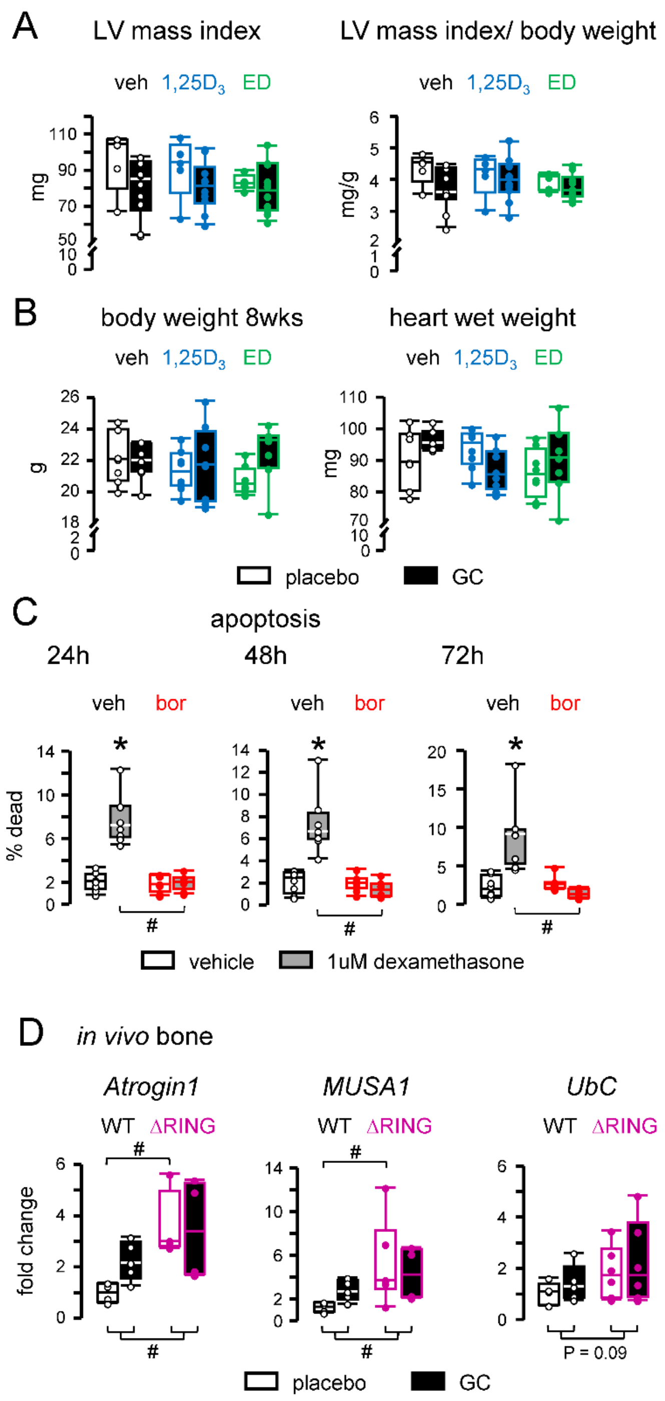

Supplemental Figure 3. (*A*-*B*) Mice were implanted with 2.1 mg/kg/d prednisolone or placebo slow-release pellets and gavaged 5x/wk with 50 ng/kg/d 1,25D3 or ED or vehicle for 8 weeks. (*A*) Left ventricular (LV) mass index absolute values and normalized by each mouse's own body weight as assessed by ultrasound echocardiography. (*B*) Whole mouse body weight and heart wet weight. *P<0.05 vs. corresponding placebo-treated, #P<0.05 vs. corresponding vehicle-treated, ^P<0.05

vs. corresponding 1,25D_3-treated by two-way ANOVA, Tukey post hoc test. ΩP<0.05 vs. placebo and vehicle-treated controls by one-way ANOVA, Dunnett's Method post hoc test. (C) Cell death was quantified by Trypan Blue uptake in OB-6 cells treated with the GC dexamethasone (dex, 1μM) ± 3nM bortezomib (Bor). n=8. *P<0.05 vs. corresponding controls, #P<0.05 vs. corresponding vehicle-treated, by two-way ANOVA, Tukey post hoc test. (D) *Atrogin1*, *MUSA1*, and *UbC* expression was quantified by qPCR in bones isolated from the indicated treated mice. N=4-5. #P<0.05 vs. corresponding WTs, by two-way ANOVA, Tukey post hoc test.

whole heart DXA

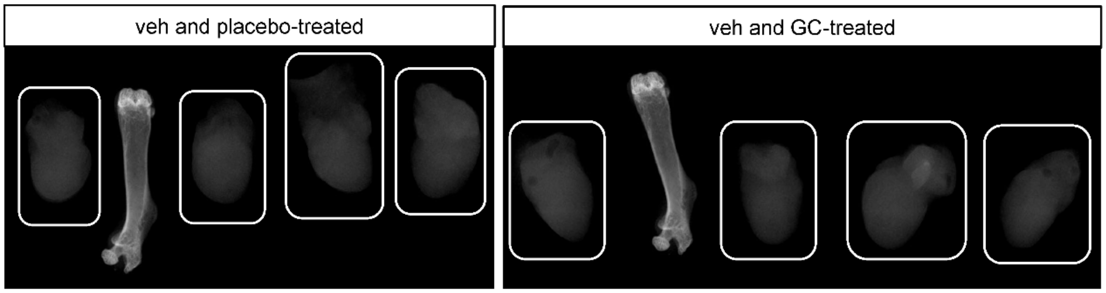

	veh and placebo-treated					veh and GC-treated				
BMD (g/cm^2)	0.0000	0.0562	0.0000	0.0000	0.0000	0.0000	0.0563	0.0000	0.0000	0.0000
BMC (g)	0.0000	0.0219	0.0000	0.0000	0.0000	0.0000	0.0214	0.0000	0.0000	0.0000

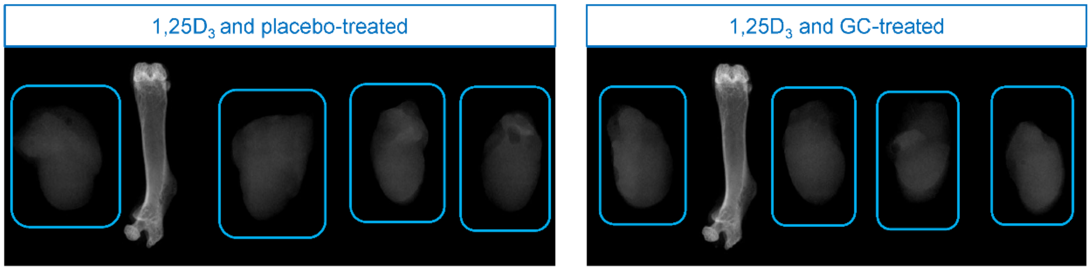

	1,25D$_3$ and placebo-treated					1,25D$_3$ and GC-treated				
BMD (g/cm^2)	0.0000	0.0563	0.0000	0.0000	0.0000	0.0000	0.0559	0.0000	0.0000	0.0000
BMC (g)	0.0000	0.0214	0.0000	0.0000	0.0000	0.0000	0.0243	0.0000	0.0000	0.0000

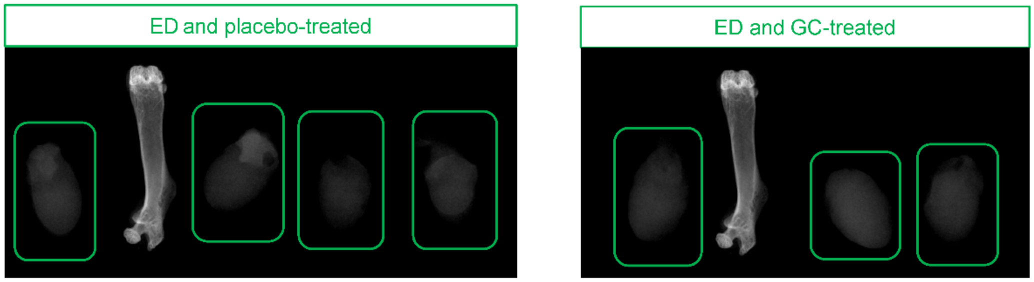

	ED and placebo-treated					ED and GC-treated			
BMD (g/cm^2)	0.0000	0.0563	0.0000	0.0000	0.0000	0.0000	0.0565	0.0000	0.0000
BMC (g)	0.0000	0.0214	0.0000	0.0000	0.0001	0.0000	0.0243	0.0000	0.0000

Supplemental Figure 4. BMD and bone mineral content (BMC) values of whole heart samples isolated from mice treated with placebo or prednisolone and vehicle or 1,25D3 or ED for 8 weeks are shown, as detected by *ex vivo* DXA scanning, n=3-4.

Circulating levels of calcium and phosphate

	placebo vehicle	GC vehicle	placebo $1,25D_3$	GC $1,25D_3$	placebo ED	GC ED
**calcium** (mg/dL) **4wks**	9.02±0.43	9.25±0.40	9.39±0.48	9.75±0.55**Ω**	12.43±0.93**#^Ω**	11.93±0.66**#^Ω**
**calcium** (mg/dL) **8wks**	9.08±1.57	9.9±0.36	10.31±0.49	10.58±0.73	13.22±1.83**#^Ω**	13.24±1.47**#^Ω**
**phosphate** (mg/dL) **4wks**	5.7±0.97	6.67±0.83*	5.84±1.04	6.54±1.24	5.52±0.72	5.03±0.90**#^**
**phosphate** (mg/dL) **8wks**	6.43±1.40	6.54±1.16	7.34±0.60	6.22±1.48	6.83±1.29	6.72±0.89

Supplemental Table 1. Mice were implanted with 2.1 mg/kg/d prednisolone or placebo slow-release pellets and gavaged 5x/wk with 50 ng/kg/d $1,25D_3$ or ED or vehicle for 8 weeks. Sera calcium and phosphate measured 4 and 8 weeks after the indicated treatments. n=10-12, *P<0.05 vs. corresponding placebo-treated, #P<0.05 vs. corresponding vehicle-treated, ^P<0.05 vs. corresponding $1,25D_3$-treated by two-way ANOVA, Tukey post hoc test. ΩP<0.05 vs. placebo and vehicle-treated controls by one-way ANOVA, Dunnett's Method post hoc test.
